# Some Amino Acids Levels: Glutamine,Glutamate, and Homocysteine, in Plasma of Children with Chronic Kidney Disease

**Published:** 2014-03

**Authors:** Fatina I. Fadel, Manal F. Elshamaa, Rascha G. Essam, Eman A. Elghoroury, Gamila S. M. El-Saeed, Safinaz E. El-Toukhy, Mona Hamed Ibrahim

**Affiliations:** 1 Department of Pediatric, Faculty of Medicine, Cairo University, Cairo, Egypt;; 2 Department of Pediatric, National Research Centre, Cairo, Egypt;; 3 Department of Clinical & Chemical Pathology, National Research Centre, Cairo, Egypt;; 4 Department of Medical Biochemistry, National Research Centre, Cairo, Egypt

**Keywords:** Glutamine, Glutamate, Homocysteine, NS, HD, Children, ESRD

## Abstract

**Background:**

The high prevalence of protein-energy malnutrition is a critical issue for patients with chronic kidney disease (CKD). Serum albumin is the most commonly used nutritional marker. Another index is plasma amino acid (AA) profile. Of these, the plasma levels of glutamine, glutamate and homocysteine, correlate well with nutritional status. We measured some plasma AAs in children with different stages CKD to provide information in monitoring the therapeutic strategy, particularly in AA supplementary therapy or protein restriction.

**Methods:**

Three amino acids were evaluated along with albumin and high sensitivity C-reactive protein (hs-CRP) in 30 patients with advanced CKD stages 4 and 5. They were divided into two groups undergoing conservative treatment (CT) (n=15) or hemodialysis (HD) (n=15). An additional group of patients with nephrotic syndrome [CKD stage 2] was also studied to assess the alterations of plasma free amino acids with the early stage of CKD. Another 30 age- and sex-matched healthy children served as controls.

**Results:**

A significant increase in plasma concentration of amino acid glutamine was observed in children with advanced CKD stages 4 and 5 when compared with controls (*P*=0.02).Plasma glutamine level was significantly higher in ESRD children on HD than in children with nephrotic syndrome (*P*=0.02). We did not find a significant difference between HD children and CT children as regard to glutamine level. Notable differences were in the plasma homocysteine level detected in the CKD groups patients, which was greater than that in controls (*P*=0.0001). Plasma homocysteine level was significantly higher in children on HD than in children with nephrotic syndrome (*P*=0.01). A significant differences was observed in hs-CRP levels between the CKD groups and the controls (*P*=0.04). Albumin levels were lower in CKD groups than in controls (*p*=0.01). Glutamine showed significant positive correlations with blood urea level (r=0.84, *P*=0.002) and blood ammonia level (r=0.72, *P*=0.0001). On multiple linear regression, urea was the only variable independently associated with an elevated plasma glutamine level (Beta=0.77, *P*=0.02).

**Conclusion:**

This study indicates that the advanced stages of CKD are associated with increased plasma concentrations of glutamine and homocysteine. Glutamine retained in the plasma of children with CRF, possibly producing higher levels of the waste products (urea and NH3). Dialysis alone is insufficient to redress completely the abnormalities in AA metabolism in ESRD children. Careful consideration of dialysis and dietary measures are necessary.

## INTRODUCTION

Nutritional and metabolic derangements, often termed as uremic malnutrition, are highly prevalent among patients with chronic kidney disease (CKD), and significantly affect the high morbidity and mortality rate observed in this patient population. The underlying mechanisms of malnutrition in CRF have not been completely clarified. Inadequate diet and a state of persistent catabolism play major roles (1). Also protein metabolism changes with loss of renal function resulting in deterioration of nutritional status (2). Altered plasma and muscle amino acid (AA) profile have also been proposed as nutritional markers. CKD patients have well-defined abnormalities in their plasma and, to a lesser extent, in their muscle amino acid profiles. Commonly, essential amino acid (EAA) concentrations are low and non-essential amino acid (NEAA) concentrations are high (3, 4). The etiology of this abnormal profile is multifactorial. Inadequate dietary intake is a major contributing factor; however, certain abnormalities occur even in the presence of adequate dietary nutrient intake, indicating that the uremic milieu has an additional effect (5). In addition, inflammation commonly seen in CKD patients has been shown to cause low plasma amino acid concentrations in CKD patients (6). Levels of plasma and intracellular amino acids are significant early indicators of protein metabolism and nutritional status assessment (2, 7). Many of the characteristic alterations in the plasma amino acid profile that are observed in chronic end-stage renal disease are already present in mild renal insufficiency. Progressive loss of renal function generally results in increasing abnormalities; these changes in plasma amino acid concentrations were usually linear with reduction in glomerular filtration rate (GFR) (8). Plasma protein and amino acid concentrations have been reported to be abnormal in patients with CRF, whether on conservative (CT) or regular dialysis treatment (9-12). These abnormalities may be related to impaired protein and amino acid metabolism, to dietary deficiencies of calories and proteins, or to amino acid and protein losses due to peritoneal dialysis or hemodialysis (13). Also increased protein degradation may be the cause of increased plasma concentration of nonessential amino acids in malnourished chronic renal patients (14, 15). Preliminary evidence indicates that metabolism of branched chain amino acid (BCAA), alanine, and glutamine is altered in end-stage renal disease (ESRD) (16, 17). Results from some laboratory (18, 19) and from other investigators (20, 21) have shown that hemodialysis (HD) induces muscle protein catabolism. The precursors for glutamine synthesis include glutamate, α-ketoglutarate, free ammonia, and amino nitrogen derived from catabolism of BCAA. Glutamine has been shown to have a regulatory effect on protein turnover (22, 23). Despite its qualitative and quantitative importance, the kinetics of glutamine in ESRD is largely unknown. Examining the metabolism of glutamine and glutamate simultaneously in CKD patients compared with normal volunteers will allow better understanding of their metabolism and its relationship to malnutrition and inflammation in CKD. Much has been studied and reported about malnutrition in advanced stages of CKD (1-3). However, there is paucity of data concerning the nutritional status in the early stages of CKD.

Homocysteine (Hcy) is thrombogenic, as it increases thromboxane formation, antagonizes nitric oxide, enhances platelet aggregation, and inhibits protein C and thrombomodulin. Hcy also is a potent mitogen for vascular smooth muscle cells (24, 25). Therefore, in end-stage renal disease, elevated plasma Hcy concentrations could contribute to the high prevalence of cardiovascular disease and the increased mortality rate (26). Hyperhomocysteinemia at an earlier stage could also accelerate progression of chronic renal disease (27).

In this study, we measured some plasma free AA (FAA) concentrations in advanced CKD (stages 4 and 5) undergoing HD or conservative treatment to detect the effect of HD on glutamine and homocysteine and compare this effect with the early stage of CKD as described by nephrotic syndrome (NS). Secondly, to provide more valuable information in monitoring the therapeutic strategy, particularly in AA supplementary therapy or protein restriction.

## METHODS

In the present study, 30 pediatric patients with advanced CKD [stages 4 and 5 based on estimated glomerular filtration rate (e-GFR) according to the National Kidney Foundation classification (28) were included in the study. They were divided into two groups undergoing CT (n=15) or HD (n=15). An additional group of age-, BMI- and gender-matched patients with nephrotic syndrome [CKD stage 2; nephrotic syndrome, *n*=15] was also studied to assess the alterations of plasma free amino acids with the early stage of CKD. They are attending the hemodialysis unit of the Center of Pediatric Nephrology and Transplantation (CPNT) and the Nephrology pediatric clinic respectively, Children’s Hospital, Cairo University, Egypt. The inclusion criteria for HD patients included a constantly elevated serum creatinine level above the normal range (ranging from 3.4 to 15.8 mg/dl) and were dialysed for not less than 6 months. They were treated with hemodialysis for 3-4 h three times weekly with a polysulfone membrane using bicarbonate-buffered dialysate. The Duration of hemodialysis was 2.82 ± 1.37 years. Patients with acute renal failure and acute on chronic renal failure were excluded from the study. Thirty age- and sex-matched healthy children were taken as controls. They were collected from the pediatric clinic (A part from the Medical Services Unit) of National Research Centre (NRC) which is one of the biggest research centres in Egypt. An informed consent was taken from all participants.The protocol of the study was read and approved by the Ethics Committee of NRC in Egypt.

The causes of ESRD on HD were nephronophthisis (n=4), posterior urethral valve (n=4), focal segmental glomerulosclerosis (n=2), unknown (n=3), atrophic solitary kidney (n=1) and nephrocystinosis (n=1). In CT patients the causes of renal failure were renal hypoplasia or dysplasia (n=1), obstructive uropathies (n=4), neurogenic bladder (n=4), not known (n=4), and metabolic (n=2). The baseline characteristics of the patients are given in Table 1. Nephrotic syndrome lesion were biopsy proven minimal change lesion (n=8), focal segmental glomerulosclerosis (n=4) or membranoproliferative glomerulonephritis (n=3).

**Table 1:** The baseline characteristics of the studied groups

Parameter	HD (n=15)	CT (n=15)	NS (n=15)	Controls (n=30)	*P*-value

Age (Years)	14.5 ± 3.21	9.14 ± 7.59	13.00 ± 1.58	10.7 ± 4.51	0.20
Gender (M/F)	6/9	5/10	8/7	7/8	0.53
BMI (kg/m^2^)	18.89 ± 3.00	17.64 ± 1.17	20.66 ± 5.12	20.60 ± 1.44	0.18
e-GFR, ml/min/1/1.73 m2	7.89 ± 2.52^a^ ^b^	15.41 ± 1.76^c^ ^d^	70.50 ± 9.19	86 ± 8.8	0.0001
Height (cm)	115.44 ± 16.86	110.32 ± 17.43	141.33 ± 11.93	150.00 ± 23.45	0.30
Weight(Kg)	22.21 ± 4.76	25.45 ± 5.85	46.60 ± 3.04	47.23 ± 5.61	0.45
Urea (mg/dL)	116.50 ± 11.88^a^ ^b^	51.12 ± 10.45^c^ ^d^	25.60 ± 8.02	7.76 ± 2.53	0.0001
Creatinine (mg/dL)	6.57 ± 1.20^a^ ^b^	3.93 ± 3.75^c^ ^d^	0.52 ± 0.27	0.73 ± 0.33	0.0001
Ammonia (μmol/l)	145.00 ± 32.25^a^ ^b^ ^e^	28.75 ± 9.07	29.00 ± 10.48	44.60 ± 4.22	0.0001

e-GFR, estimated glomerular filtration rate. HD, hemodialysis; CT, conservative treatment; NS, nephrotic syndrome; BMI, Body mass index.

a *P*<0.001 vs. controls and HD;

b *P*<0.001 vs. HD and NS;

c *P*<0.001 vs. controls and CT;

d *P*<0.001 vs. CT and NS;

e *P*<0.001 vs. HD and CT.

Five milliliters of heparinized venous blood samples was collected from the patients and healthy subjects in the morning, after an overnight fast. The samples were centrifuged at 2000 rpm for 15 min, and the plasma separated and stored in vials at -80°C until analysis. The samples were processed for glutamine, glutamate, homocysteine, urea, creatinine, albumin and C-reactive protein (CRP). Plasma urea, creatinine and albumin were made by using an Olympus AU400 (Olympus America, Inc.,Center Valley, Pa., USA). hs- CRP was performed by solid-phase chemiluminescent immunometric assay (Immulite/Immulite 1000; Siemens Medical Solution Diagnostics, Eschborn, Germany) (29). Detremination ammonia levels was done by colorimetric method (Biodiagnostic Comp.) (30).

Quantification of glutamine and glutamate in plasma by tandem mass spectrometry. Principal: Once a sample containing the molecule of interest has been introduced into the mass spectrometer either directly or after some kind of sample preparation or separation, the sample is ionized, positively or negatively charged fragment ions are generated, sorted by their mass-to-charge (m\z) ratio and recorded. During standard ESI, the sample is dissolved in a polar, volatile solvent and pumped through a narrow, stainless steel capillary (75-150 micrometers i.d.) at a flow rate of between 1 μl\min and 1 ml\min. Specifications: The HPLC-ESI-MS system consisted of an HP 1100 Series HPLC instrument (quaternary pump and degasser, column compartment, and auto-sampler) and an LCQ ADVANTAGE MAX mass spectrometer from Thermo Finnigan. “X calibure 1.4” software. Methodology: Amino acids were extracted with methanol from 2 micro of plasma, adsorbed on a 3- mm filter-paper disk for 15 min. The amino acids were treated with 240 (µ)L of dimethylacetaldimethyl formamide- acetonitrile-methanol (2:5:5 by volume) at room temperature for 5 min, excess reagents were evaporated, and the residue was treated with isobutanol-mol\L-hydrogen chloride (200 μL) at room temperature for 10 min and then was evaporated to dryness. The derivatives were dissolved in 2 ml of acetonitrile-water-formic aid (50:50:0.025 by volume). Sample were injected from a 96-well tray at 2-min intervals. Muliple-reaction monitoring experiments with six ion pairs, representing a neutral loss of 73 atomic mass units (AMU, for glutamine) and a neutral loss of 102 AMU (for glutamic acids) (31).

Quantitative determination of homocysteine level in serum. The Diazyme homocysteine Enzymatic assay is based on a novel assay principle that assesses the co-substrate conversion product instead of assessing co-substrate.The concentration of homocysteine in the sample is indirectly proportional to the amount of NADH converted to NAD+. The Kit was supplied from Diazyme laboratories (USA) catalog No. DZ568A (32).

### Statistical Analysis

All values obtained were expressed as mean ± standard deviation of mean (SD). Mann Whitney U test was performed to compare the difference in the means between the controls and the study groups. A *P*-value <0.05 was considered as statistically significant. Spearman’s rank correlation analysis was done to study the correlation between different variables and amino acids. Multiple linear regression analysis using the stepwise method was performed to determine the contribution of various factors as independents or covariates to glutamine as the dependent variable. Statistical analysis was performed using SPSS for Windows Version 16.

## RESULTS

As shown in Table 2, a significant increase in plasma concentration of amino acid glutamine was observed in children with advanced CKD stages 4 and 5 when compared with controls (*P*=0.02). Plasma glutamine level was significantly higher in ESRD children on HD than in children with nephrotic syndrome (*P*=0.02).We did not find a significant difference between HD children and CT children as regard to glutamine level. No significant difference was observed in glutamate level between CKD groups and the controls (*P*=0.3). Notable differences were in the plasma homocysteine level detected in CKD groups, which was greater than that in control samples (*P*=0.0001). Plasma homocysteine level was significantly higher in ESRD children on HD than in children with NS, (*P*=0.01). A significant differences was observed in hs-CRP levels between all CKD groups and the controls (*P* = 0.04).Albumin levels were lower in all CKD groups than in controls (*p*=0.01). No significant correlation was observed between albumin and amino acids gulamine, glutamate and homocysteine in the CKD patients (study groups). Among the studied AA, only homocysteine showed a positive correlation with hs-CRP (r=0.67, *P*=0.002). Glutamine showed significant positive correlations with blood urea level (r=0.84, *P*=0.002) and blood ammonia level (r=0.72, *P*=0.0001). There were no any correlations between plasma glutamate level and other individual variables. On multiple linear regression, urea was the only variable independently associated with an elevated plasma glutamine level (Beta=0.77, *P*=0.023).

**Table 2:** Mean, standard deviation (SD) and P-value for biochemical parameters among chronic kidney disease patients (study groups) and the controls

Parameter	HD (n=15)	CT (n=15)	NS (n=15)	Controls (n=30)	*P*-value

Glutamine (μmol/l)	376.47 ± 106.01^a^ ^b^	329.52 ± 33.35^c^ ^d^	250.14 ± 34.47	248.04 ± 45.25	0.0001
Glutamate (μmol/l)	65.88 ± 4.14	58.02 ± 7.65	62.12 ± 8.99	72.00 ± 10.11	0.21
Homocysteine (μmol/l)	26.55 ± 8.33^a^ ^b^	22.00 ± 4.06^c^	15.80 ± 1.48^e^	8.60 ± 1.14	0.0001
Albumin (g/dL)	3.76 ± 0.47^a^	3.89 ± 0.56^c^	3.10 ± 0.78^e^	4.92 ± 0.39	0.01
hs-CRP (mg/dL)	5.00 ± 1.16^a^	3.04 ± 3.24^c^	4.2 ± 1.9^e^	1.35 ± 0.65	0.04

hs-CRP, High sensitivity C-reactive protein.

a *P*<0.05 vs. controls and HD;

b *P*<0.01 vs. HD and NS;

c *P*<0.001 vs. controls and CT;

d *P*<0.001 vs. CT and NS;

e *P*<0.05 vs. controls and NS.

## DISCUSSION

Patients with ESRD on maintenance dialysis have a number of abnormalities of protein metabolism, including increased catabolism, reduced synthesis, muscle wasting, negative nitrogen (N) balance, increased production of toxic metabolites, and characteristically abnormal intracellular and plasma FAA concentrations (33-35). Rates of protein synthesis in normal and pathologic conditions are more closely related to the intracellular amino acid pool than to plasma amino acid levels. However, plasma amino acid levels may directly reflect disturbances of protein or amino acid metabolism and interorgan exchange in the uremic state (36, 37).

The present study showed a significant increase in plasma concentration of amino acid glutamine in children with the advanced stages of CKD when compared with controls. Plasma glutamine level was significantly higher in ESRD children on HD than in children with NS. No significant difference was found between children on HD and the CT group as regard to glutamine. This result may indicate that dialysis alone is insufficient to redress completely the abnormalities in protein catabolism and protein synthesis in ESRD children .The excess FAAs accumulated in plasma might indicate a higher rate of protein catabolism in patients undergoing maintenance dialysis rather than decreased urinary excretion (38, 39). Enhanced protein breakdown by acidosis points to the physiological changes in patients on dialysis (40). There is increasing evidence that disturbances in AA metabolism may result from alterations in extra- and intracellular compartments, especially interorgan transport by erythrocytes, and as a result of muscle degradation (41). Hormonal derangements and alterations in intermediary metabolism may also be related to abnormalities of AA metabolism (42).

While dialysis can diminish or ameliorate uremia symptoms, it does not correct the metabolic disturbances and catabolism. Chaung *et al*. (7) reported that a 17.9% reduction in total FAA concentration from pre-HD to post-HD samples. There was a wide variation in most individual FAAs after HD, whereas glutamic acid, isoleucine, leucine, tryptophan, and hydroxylysine levels either increased or remained stable. There were, however, only a few individual FAAs that differed significantly between pre-HD and post- HD samples. While there was a 17.9% reduction in total FAA concentration after HD, the total FAA level in post-HD samples remained 25.4% higher than in control samples. Maintenance dialysis thus partially eliminates the FAAs accumulated in plasma, but the correction of FAA abnormalities seems to be limited. For this reason, attention should still be paid to restriction of protein intake and supplementation with AA mixtures (with proper proportions of EAAs and NEAAs), as this may have the beneficial effects of increasing body weight and normalizing both the plasma and intracellular amino acid profile (40, 41).

Malnutrition may be one of the causes of AA abnormalities detected in these HD patients, as evidenced by the significant lower weight in HD patients compared to the control group. This is in accordance with Sen and Prakash (40), who found that malnutrition is a common clinical problem in dialysis patients, which is multifactorial in origin, and Magorzewicz *et al*. (41) who reported that despite quite good nutritional status, dialyzed patients have abnormalities in their AA profiles.

No significant difference was found between NS and the controls as regard to glutamine. This result may be due to small number of NS patients. Kumar *et al*. (38) concluded that malnutrition seen in early stages of CKD patients in the form of hypoalbuminemia and decreased concentrations of BCAA points to the need to evaluate the nutritional status in the early stages itself. Also, El-Gayar *et al*. (39) had reported that a highly significant rise of plasma glutamic acid and hydroxyproline was detected in nephrotics and these changes in AA levels occurred before the rise in serum creatinine.

Increased postabsorptive plasma glutamate levels have been linked to conditions with loss of body cell mass (BCM); in a study of 60 cancer patients an increased plasma glutamate level was attributed to a decreased capacity of peripheral muscle uptake as the uptake of glutamate and glucose over the leg were lower than in age-matched control subjects (43). In addition, BCM determined with impedance measurements was reported to decrease with increasing plasma glutamate levels in a longitudinal study with two healthy subjects (44).

Plasma and whole blood glutamate levels have been shown to be significantly elevated in patients with chronic renal failure (CRF) (18, 41). Insulin like growth factor binding protein (IGFBP) is elevated in patients with end stage renal failure, resulting in reduced bioactivity of insulin like growth factor (IGF-1) (45). IGF-1 has been shown to significantly decrease blood glutamate levels. This effect is more pronounced in healthy patients compared with patients suffering from chronic renal failure (CRF) (44). Elevated levels of IGFBP promote elevation of plasma glutamate levels. CRF, which is known to be associated with elevated levels of IGFBP, results in very high concentrations of glutamate in both plasma and whole blood of the patients with CRF. In conditions involving wasting of muscle mass, muscle glutamate has been shown to be the only amino acid to inversely correlate with RBC glutamate. Blood glutamate remains elevated in patients with CRF and has been shown to decrease following hemodialysis, correlating with a decrease in urea (46). Thus, elevated glutamate levels found in blood of patients with CRF and ARF may contribute to uremic encephalopathy (47, 48).

Urea is the major metabolic end product of amino groups derived from amino acids. One nitrogen atom of the urea molecule is supplied by free NH3 and the other by aspartate. Glutamate is the immediate precursor of both the ammonia and aspartate nitrogen. In our study, the concentrations of plasma FAAs involved in urea cycle metabolism including glutamine, as well as NH3, were significantly higher than controls in children with ESRD and Glutamine showed significant positive correlations with blood urea level and blood ammonia level. These abnormally high levels will produce more urea in the blood and promote the formation of NH4, contributing to the hyperammonemia often seen in these patients (7). However, no episodes in advanced liver failure and encephalopathy were found in our patient children. In addition, reduced urinary excretion of urea and NH3 may also contribute to the high blood urea and NH3.

We found increased concentrations of sulfur-containing amino acids, Hcy, in patients with CKD when compared with controls. Evidence is accumulating that increased tHcy is implicated in the pathogenesis of vascular damage (25). Folic acid is a potent tHcy-lowering agent that has been shown to induce a 16% to 40% decrease in tHcy in patients on dialysis (26, 27). Chuang *et al*. (7) stated that in order to normalize serum concentrations of Hcy, they routinely treated nearly all patients on dialysis in their study with folic acid (5 mg). They begin this treatment when chronic renal failure develops. Even though the Hcy in the HD and peritoneal dialysis (PD) samples was slightly too moderately increased, it probably does not contribute to atherogenesis in these patients. NS is associated with increased risk of atherosclerosis as proteinuria and hyperhomocysteinaemia are independently associated with increased risk of atherosclerosis and cardiovascular disease (49).

Serum albumin was measured as a nutritional marker. A significant decrease in albumin levels were observed in CKD patients compared with the control group. Hypoalbuminemia in advanced CKD patients may reflect non-nutritional factors, such as external losses and decreased synthesis (6). There is a concern over the appropriateness of serum albumin concentrations to assess nutritional status in CKD patients, especially if confounding factors such as inflammation are not taken into account. Also, the systemic inflammatory response stimulates protein catabolism (5). Amino acids released from muscle protein provide a substrate for the synthesis of acute phase proteins and proteins of the immune system (9). In our study, we evaluated CRP levels as a marker of inflammation. Among the studied AA, only homocysteine showed a positive correlation with CRP. Vieira *et al*. (50) found a positive correlation between tHcy levels and markers of inflammation, namely, IL-6 and TNF-α. They found that inflammation and malnutrition was associated with higher levels of tHcy.

In conclusion, this study indicates that the advanced stages of CKD are associated with increased plasma concentrations of some AA due to increased protein catabolism. The accumulation of glutamine resulted in more waste products, such as urea and ammonia, potentially contributing to high morbidity in HD patients. Dialysis alone is insufficient to redress completely the abnormalities in protein catabolism and protein synthesis in ESRD children. Therefore, judicious use of dietary measures may still require.
